# Overview of Movement Disorders Secondary to Drugs

**DOI:** 10.3390/clinpract13040087

**Published:** 2023-08-18

**Authors:** Jamir Pitton Rissardo, Nilofar Vora, Bejoi Mathew, Vikas Kashyap, Sara Muhammad, Ana Letícia Fornari Caprara

**Affiliations:** 1 Neurology Department, Cooper University Hospital, Camden, NJ 08103, USA; 2 Medicine Department, Terna Speciality Hospital and Research Centre, Navi Mumbai 400706, India; nilofar031202@gmail.com; 3 Medicine Department, Sri Devaraj Urs Medical College, Kolar Karnataka 563101, India; bejoimath@gmail.com; 4 Medicine Department, Vardhman Mahavir Medical College and Safdarjung Hospital, New Delhi 110029, India; vikaskashyap346@gmail.com; 5 Neurology Department, Mayo Clinic, Rochester, MN 55906, USA; muhammad.sara@mayo.edu; 6 Medicine Department, Federal University of Santa Maria, Santa Maria 97105-900, RS, Brazil; ana.leticia.fornari@gmail.com

**Keywords:** drug-induced, aims, parkinsonism, tardive syndromes, antipsychotics, anticonvulsants, illicit, neuroleptics, dopamine receptor blocker agents, dyskinesia

## Abstract

Drug-induced movement disorders affect a significant percentage of individuals, and they are commonly overlooked and underdiagnosed in clinical practice. Many comorbidities can affect these individuals, making the diagnosis even more challenging. Several variables, including genetics, environmental factors, and aging, can play a role in the pathophysiology of these conditions. The Diagnostic and Statistical Manual of Mental Disorders (DSM) and the International Statistical Classification of Diseases and Related Health Problems (ICD) are the most commonly used classification systems in categorizing drug-induced movement disorders. This literature review aims to describe the abnormal movements associated with some medications and illicit drugs. Myoclonus is probably the most poorly described movement disorder, in which most of the reports do not describe electrodiagnostic studies. Therefore, the information available is insufficient for the diagnosis of the neuroanatomical source of myoclonus. Drug-induced parkinsonism is rarely adequately evaluated but should be assessed with radiotracers when these techniques are available. Tardive dyskinesias and dyskinesias encompass various abnormal movements, including chorea, athetosis, and ballism. Some authors include a temporal relationship to define tardive syndromes for other movement disorders, such as dystonia, tremor, and ataxia. Antiseizure medications and antipsychotics are among the most thoroughly described drug classes associated with movement disorders.

## 1. Introduction

Prescribed and illicit drugs can cause adverse neurological effects such as movement abnormalities. Dopamine-receptor-blocking agents, such as antipsychotics and antiemetics, are the most prevalent causes of drug-induced movement disorders [1]. In this context, abnormal movements secondary to drugs can range from tremors to life-threatening emergencies. The abnormal movements can be categorized as acute, subacute, or tardive syndromes based on the onset of the drug to the beginning of the movement disorder. Acute drug-induced movement abnormalities can occur minutes to days after the administration of the offending drug. Among them are akathisia, tremor, neuroleptic malignant syndrome, serotonin syndrome, parkinsonism, and acute dystonic symptoms. Subacute drug-induced movement disorders can occur within days to weeks following drug initiation. Tardive medication-induced movement syndromes develop after exposure to an offending drug or within weeks of drug discontinuation [2,3].

Movement disorders are characterized as hyperkinetic or hypokinetic based on their major phenomenology. Tremor, dystonia, chorea, myoclonus, tics, and akathisia are hyperkinetic syndromes characterized by excess movement. The decreased movement in hypokinetic disorders unrelated to weakness or paralysis characterizes parkinsonism [4]. There are several proposed classification systems for the categorization of drug-induced movement disorders, but none include all types of drug-related abnormal movements. Thus, many patients with drug-induced movement disorders are probably misclassified [5]. The Diagnostic and Statistical Manual of Mental Disorders (DSM) and the International Statistical Classification of Diseases and Related Health Problems (ICD) are among the most commonly utilized to classify symptoms in electronic medical records (Table 1) [6].

When properly managed, patients with movement disorders secondary to drugs can achieve full remission. Nevertheless, these disorders are often underrecognized in clinical practice, which could negatively impact the quality of life of patients and their caregivers for years [7]. Although they were primarily described with antipsychotic medications, several pharmacological substances were reported with movement disorders. Drug-induced movement disorders are a worldwide issue and usually occur while managing psychiatric and gastrointestinal motility disorders [8]. This is often the case because of the scarcity of medications that do not affect the nigrostriatal pathway. Acute antipsychotic-associated movement disorders, such as akathisia and parkinsonism, occur shortly after the initiation of the medication or titrations, and chronic antipsychotic-related movement disorders, such as tardive dyskinesia and tardive dystonia, usually occur after long-term exposure to antipsychotics [9]. Early identification and treatment increase the likelihood of mitigating adverse outcomes.

Differential diagnosis between the idiopathic nature of the disorder and the secondary cause from a drug could be challenging in clinical practice. The most common approach to the occurrence of a side effect is the discontinuation of the offending drug, but sometimes the medication causing the adverse effect is essential for the management of the patient. Thus, the significance of studying side effects and how to best manage them with dose adjustment may be great for patients with difficult and uncontrolled primary disease and comorbidities. The differential diagnosis of drug-induced movement disorders should always include functional movement disorders, which can present with any type of abnormal movement [10]. It is noteworthy that the most common presentation of functional movement disorder overlaps with those secondary to drugs, in which tremor represents the most common clinical manifestation, followed by dystonia, myoclonus, and gait disorders [11]. The distinction between drug-induced movements and functional movement disorders is based on two main characteristics: inconsistency and incongruence [12].

Basal ganglia are a diversified array of linked nuclei that play a significant role in movement execution and signal relay. The classical paradigm suggests a direct (striatonigral) and indirect (striatopallidal) pathway inside the basal ganglia, including striatal projection neuron subpopulations, in which GABA plays an important role in the modulation of cortical impulses. Moreover, the cortical pathways can alter dopamine-dependent signaling, increasing or decreasing locomotor activity [13]. These pathways’ selective contributions have been demonstrated in mice with dopamine- and cAMP-regulated phosphoprotein Mr 32 kDa (DARP-32) deletion in nigrostriatal neurons in response to tropane alkaloids, such as cocaine [14].

Several neurotransmitter systems may be associated with the development of movement disorders. Any of these changes may result in impairments of the motor and cognitive systems. In this context, the dopaminergic system is distributed all across the cortical and subcortical regions. The D1 and D2 receptors are the most abundant in basal ganglia cells, with other receptor subtypes being less expressed. Dopamine-receptor-containing medium spiny neurons express specific receptor subtypes in their projections. The striatonigral circuit expresses D1 receptors selectively, whereas the striatopallidal pathway expresses D2 receptors. D1, D2, and D3 receptors are primarily responsible for locomotor control [15]. Serotonin regulates extrapyramidal motor activity via serotonin receptors in numerous cortical locations and the striatum. There are several subtypes of serotoninergic receptors, which have distinct distribution patterns. For example, 5HT1F and 5HT3A are absent in the caudate, substantia nigra, or globus pallidus. However, others have moderate or low expression levels in the same locations in marmoset brains [16].

The serotoninergic system more commonly regulates cognition to the detriment of motor functions than the dopaminergic system. Experimental animal models show that serotonin might influence dopaminergic motor performance in the nigrostriatal system. GABA is the primary inhibitory transmitter in the central nervous system and can modulate motor function by activating the GABAA receptor. More than sixteen receptor subunits of neurotransmitters, which play a significant role in motor control, have been found across the central nervous system [16,17].

## 2. Methods

We searched six databases to locate existing reports on movement disorders secondary to drugs published until June 2023 in electronic form. Excerpta Medica (Embase), Google Scholar, Latin American and Caribbean Health Sciences Literature (Lilacs), Medline, Scientific Electronic Library Online (Scielo), and Science Direct were searched. Search terms were “parkinsonism, tics, dyskinesia, dystonia, stuttering, myoclonus, restless legs syndrome, akathisia, tremor, chorea, restlessness, ataxia, ballism, hyperkinetic, hypokinetic, bradykinesia, movement disorders”. These terms were combined with “drug-induced, medication-induced” (Table 2).

## 3. Akathisia

**Figure 1:**
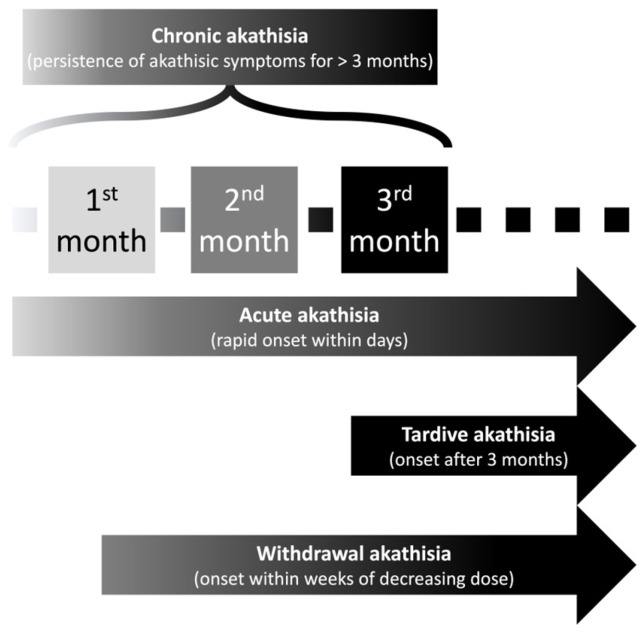
Classification of antipsychotic-induced akathisia according to the time of onset in the course of antipsychotic treatment.

Akathisia is a neurological disease characterized by a sense of inner restlessness and difficulty sitting or standing still, with a prevalence of up to thirty percent with antipsychotic use [18]. The symptoms of akathisia can range from mild to severe. In mild cases, people may feel restless or fidgety. In more severe cases, patients report a sense of loss of control over their bodies. People suffering from akathisia may feel compelled to continuously move, fidget, pace, or rock back and forth. They may also struggle to concentrate or sleep [19]. Akathisia can be very distressing and can interfere with daily activities. Akathisia may be generalized akathisia pacing or focal akathisia, often reported as pain or discomfort [20]. Sachdev et al. designed an interesting classification system specific to antipsychotic-induced akathisia according to the time of onset in the course of antipsychotic treatment. The classification system includes acute akathisia, tardive akathisia, withdrawal akathisia, and chronic akathisia (Figure 1) [21].

Some medications reportedly associated with akathisia are antipsychotics, antiemetics, and some antidepressants [22]. Notably, akathisia can be a symptom of a neurological disorder and is challenging to differentiate from agitation in people suffering from Huntington’s disease, for example. Akathisia has been largely overlooked and undiagnosed in clinical practice. Conditions that do not fulfill the diagnostic criteria for akathisia could still benefit from therapeutical akathisia measures but are largely underdiagnosed, probably due to the subjective component of its diagnosis [23]. Moreover, patients with akathisia frequently do not complain to their primary care providers about their symptoms due to fear of being labeled with a psychiatric or functional condition. Jouini et al. reported that the delay between drug-induced akathisia onset and its diagnosis is 7.1 years (standard deviation = 8.8) [24].

Gamma-aminobutyric acid (GABA) hypoactivity, noradrenergic hyperactivity, and serotonergic dysfunction may all play a role in the etiology of neuroleptic-induced akathisia [25]. Notably, the pathophysiology of akathisia can be categorized into hyper-dopaminergic and hypo-dopaminergic [26]. Treatment may involve discontinuing the offending drug as soon as it is diagnosed or decreasing the prescribed dose. Another therapeutic option is to switch to atypical antipsychotics, such as clozapine, olanzapine, or quetiapine, or to add a beta-blocker, anticholinergic, or mianserin [27]. It is worth mentioning that Laoutidis et al. demonstrated the efficacy of 5-HT2A receptor antagonists in treating neuroleptic-induced akathisia in a meta-analysis [28].

## 4. Dystonia

Dystonia is a movement disorder characterized by involuntary muscular contractions that can result in twisting or repetitive movements. The symptoms of acute dystonia typically appear within hours or days of exposure to the offending agent. They can affect any muscle group in the body, but the neck, face, and limbs are the most commonly involved areas [29]. Dystonia can be mild or severe and be accompanied by other symptoms, such as difficulty speaking or swallowing [30]. The abnormal postures in patients with dystonia may be misdiagnosed as functional, since dystonia often occurs in patients with neuropsychiatric diseases, and it can be mistakenly identified as catatonia [31,32]. In most cases, acute dystonia is a self-limited condition that resolves within a few days or weeks [33]. However, in some cases, it can be more persistent. A peculiar finding in drug-induced dystonia, compared to the other movement disorders associated with drugs, is a tendency to occur in younger patients and individuals of Asiatic origin [34].

The most common clinical classification of drug-induced dystonia in the literature is according to body regional distribution. There are five presentation types: focal, segmental, multifocal, generalized, and hemidystonia [35]. Focal dystonias only affect one body region and can be reported as blepharospasm, oromandibular, cervical, laryngeal, and limb dystonia. Interestingly, cranial dystonia is defined as a subtype of dystonia affecting the lower facial muscles, jaw, or tongue in the presence of blepharospasm [36]. Notably, some authors include severe acute dystonic reactions in drug-induced dystonia, such as oculogyric crisis (paroxysmal, conjugate, and typically upward deviation of the eyeball) and dystonic opisthotonus (spastic contraction of the extensor muscles of the neck, trunk, and lower extremities leading to backward arching from neck to heels) [37].

Dopamine-blocking drugs, such as first-generation, highly potent antipsychotics including haloperidol, fluphenazine, and pimozide, are more commonly associated with dystonia (Table 3) [38]. In contrast, less potent antipsychotics such as chlorpromazine and thioridazine have a lesser tendency to cause it, probably because of their inherent anticholinergic effects [39]. Acute dystonia is also a side effect of atypical antipsychotics, although the exact mechanism is uncertain. Rivastigmine and bupropion have also been linked in case studies to acute dystonic reactions [40]. It is worth mentioning that stroke, traumatic brain injury, or infection can also cause dystonia [41]. Another possible hypothesis explaining drug-induced dystonia is regarding GABAergic neurotransmission [42]. The decreased GABA concentrations can lead to an interruption in the direct and indirect pathways, probably predominantly affecting the indirect pathway. As a result, this disruption can increase the thalamocortical drive and eventually cause dystonia [43]. Treatment for acute dystonia typically involves discontinuing the offending agent and administering medications that block the effects of dopamine. A therapeutical option already described in the literature is antihistaminic therapy, such as procyclidine and diphenhydramine [44]. Early diagnosis and appropriate treatment are important to prevent possible complications.

## 5. Myoclonus

Myoclonus is a rapid, abrupt, short, “shock-like”, involuntary movement elicited by either a sudden, brief break in the contraction of active postural muscles (also known as “negative myoclonus” or “asterixis”) or by a muscular contraction (in the case of “positive myoclonus”). The most common description of myoclonus is based on the affected body parts, which include focal, multifocal, and generalized myoclonus [46]. One important clinical presentation of myoclonus is unexplained occasional falls, which were already reported with myoclonus secondary to drugs [47]. Drug-induced myoclonus is a movement disorder that is insufficiently assessed in the literature. Most authors do not describe important electrophysiological characteristics supporting the diagnosis of the myoclonus neuroanatomical source. A precise report of drug-induced myoclonus should appreciate the myoclonus response to stimulus, electroencephalogram, and electromyography [48]. In this way, it is possible to approximately differentiate between a subcortical and cortical origin, the most common sources of myoclonus secondary to drugs (Table 4) [49].

Levodopa, tricyclic antidepressants, and bismuth salts can induce myoclonus (level II evidence) [50,51]. However, the evidence for many other medications in a cause and effect relationship is less substantial. Although the pharmacological processes causing this detrimental impact are unknown, enhanced serotonergic transmission may be implicated in the development of myoclonus by various medications [52]. Drug-induced myoclonus normally resolves once the offending substance is discontinued, although particular therapies are required in certain circumstances. One of the most common therapeutical approaches is prescribing benzodiazepines to shorten the recovery time [53].

## 6. Parkinsonism

Most authors believe that drug-induced parkinsonism (DIP) is the most common drug-induced movement disorder [54]. However, this statement should be critically analyzed because it might be a misconception that does not reflect reality accurately. In fact, when objectively analyzing the published literature, the most commonly reported movement disorders associated with medications probably are ataxia and postural/action tremor, frequently found with some medications in more than ten percent of users. One possible explanation for this finding is that most clinical trials assess tremors and ataxic symptoms with standard questionnaires, in which a higher frequency of false-positive responses could be found.

DIP was recognized in the early 1950s with antipsychotic drugs. It was reported that DIP occurs during the initial period of antipsychotic medication exposure [55]. The pathophysiology of DIP is related to disruptions in the basal ganglia motor pathways secondary to dopaminergic receptor blockade [56]. The clinical features of DIP are variable, with patients predominantly presenting with bradykinesia and rigidity. Therefore, DIP can mimic Parkinson’s disease (Table 5) [57,58]. To differentiate DIP from Parkinson’s disease, dopamine transporter imaging is useful to compare the symmetry of radiotracer uptake in the striatum [59]. Several studies have demonstrated an individual variation in the possibility of extrapyramidal side effects with dopamine receptor antagonist drugs. It has been reported that elderly patients are more susceptible to DIP, presumably due to the decreased neuronal circuits and striatal dopamine accompanying aging [60]. Patients with DIP present with a motor syndrome of bradykinesia, rigidity, and/or resting tremor that is clinically indistinguishable from idiopathic Parkinson’s disease.

In individuals with Parkinson’s disease, there is asymmetric striatal neuronal damage, which is more significant contralateral to the affected side and involves the posterior putamen more than the anterior putamen and caudate. SPECT and PET neuroimaging can demonstrate these findings, but the quality and resolution of PET imaging are better than those of SPECT. On the other hand, patients with DIP do not develop abnormalities in the striatal region, and the radiotracer will be observed in both basal ganglia regions [61]. Some radiotracers already studied in individuals with probable drug-induced parkinsonism are I-123 FP-CIT (Ioflupane), I-123 βCIT, F-18 FP CIT, Tc99m TRODAT, and F-18 DOPA. It is worth mentioning that there are significant limitations to these procedures that include the requirement of thyroid blockers and the availability of this technology [3].

Most patients will recover from DIP within a few weeks of discontinuing the offending drug, but the motor symptoms may sometimes persist. Some predictors associated with a full recovery from DIP are acute–subacute onset, the absence of non-motor symptoms, a normal brainstem auditory evoked response, and DIP unrelated to calcium channel blockers [62]. The first step in management involves gradual cessation of the offending drug and, if needed, replacing typical antipsychotic agents with atypical antipsychotics, especially clozapine. It has also been shown that anticholinergics such as benztropine and trihexyphenidyl can be used to treat DIP with predominant rigidity [63].

The main finding of DIP is symmetrical rigidity, which some authors describe as mainly being akinetic. In this context, several potentially life-threatening conditions can present with hypokinetic movement disorders. Parkinsonism-hyperpyrexia disorder, serotonin syndrome, and neuroleptic malignant syndrome should be ruled out in the acute–subacute onset of drug-induced parkinsonism (Table 6) [64].

## 7. Tremor

A tremor is defined as an involuntary rhythmic oscillatory movement. It can be classified according to clinical characteristics and etiology. Tremor syndromes consist of isolated tremors or tremors combined with other clinical features. Tremors may be further classified based on the behavior in which they occur. These are action tremors, intention tremors, and resting tremors. Action tremors vary widely in amplitude and frequency (4–12 Hz) and occur with maintained posture or movement. An intention tremor is a kinetic tremor (typically < 5 Hz) that occurs during the terminal part of a target-directed action and is larger in amplitude. On the other hand, resting tremors occur in limbs supported against gravity and are usually 4–6 Hz. These types of tremors decrease with movement.

Pathophysiological studies suggest that all types of tremors are associated with overlapping cerebral networks. These involve the basal ganglia and the cerebello-thalamo-cortical circuit. Tremors are considered by some authors the most difficult movement disorder for the clinician to correctly diagnose and classify because there are many causes of tremors. One of the causes usually seen in clinical practice is drugs. Drugs can cause tremors and may exacerbate them, especially in individuals with fine postural or kinetic tremors (Table 7). Commonly implicated drugs with tremors are caffeine and beta-adrenergic agonists. Other drugs known to cause tremors are amiodarone, selective serotonin (and norepinephrine) reuptake inhibitors, amitriptyline, lithium, valproate, dopamine receptor agonists, vesicular monoamine transporter (VMAT) 2 inhibitors, or drugs of abuse. Knowledge of drug-induced tremors aids in obtaining a prompt diagnosis, avoiding unnecessary tests, and, at the same time, providing the most suitable treatment.

Some criteria should always be observed in individuals presenting with probable drug-induced tremors: the exclusion of other medical causes of tremor; the temporal relation of the tremor to the start of therapy with the medication; and a dose–response relation with increased tremor coincident with increased medication dose and a lack of tremor progression over time. Once a tremor is attributed to drugs, some acceptable therapeutical options are decreasing the dosage of the drug, discontinuation of the drug, or replacement of the drug by a different agent. Additionally, depending upon the acceptability of the side effects and the risk–benefit ratio, the drug may be continued, and the decision needs to be individualized. Sometimes, beta-blockers are helpful in cases where the drug needs to be continued or discontinuation of the drug does not result in full improvement immediately [77].

## 8. Tardive Syndromes and Dyskinesias

Tardive dyskinesia is a subgroup of tardive syndromes. Tardive dyskinesia is a prevalent drug-induced movement disorder that manifests with stereotypical movements accompanied by dystonia, choreoathetoid movements, tremors, myoclonus, and akathisia. The term “classic tardive dyskinesia” represents oro-bucco-lingual stereotypic movements [78]. Among the most common risk factors for tardive dyskinesia is long-term exposure to high-level dosages of antipsychotic drugs [79]. Other predisposing factors include African American ethnicity, older age, diabetes mellitus, and the co-occurrence of dystonia or parkinsonism [80].

The pathophysiology of tardive dyskinesia is related to hypersensitivity in dopamine receptors and structural dysfunction in the neuronal activity of GABAergic and N-methyl-D-aspartate (NMDA) receptors [81]. Identifying drug-induced tardive dyskinesia requires the fulfilment of certain criteria, such as abnormal, involuntary movements that must occur in two or more body parts. Motor symptoms occur at least three months after starting treatment with an antipsychotic medication. No other conditions should explain the abnormal movement patterns. It is worth remembering that there are several classification systems for tardive dyskinesias. One example is the classification system only including antipsychotics with tardive syndrome. On the other hand, some authors include all classes of medications to be associated with tardive syndromes [82].

Antipsychotics, antidepressants, mood stabilizers, and anti-parkinsonian drugs can cause tardive syndromes. In this context, it is recommended to monitor patients with antipsychotic therapy for the incidence of tardive dyskinesia every three to twelve months. The first-line treatment for tardive dyskinesia is vesicular monoamine transporter 2 (VMAT2) inhibitors (tetrabenazine, valbenazine, deutetrabenazine) [83].

### Levodopa-Induced Dyskinesia

One possible cause of dyskinesia in people with Parkinson’s disease is levodopa-induced dyskinesia. However, it is also important to consider that dyskinesias can occur with the progression of the neurodegenerative disorder in itself due to dopaminergic neuronal loss [84]. A young age at Parkinson’s disease onset, higher levodopa dose, low body weight, and more advanced disease are risk factors for the development of levodopa-induced dyskinesia [85]. To change the levodopa dosage, it is important to establish a pattern in the time and duration of dyskinesias. Depending on how long the dyskinesia has been present, the levodopa dosage can often be decreased while maintaining effectiveness. A modification in levodopa dosage may not be necessary since patients will most likely not be affected in their activities of daily living because of minor dyskinetic symptoms. One of the options in the management of levodopa-induced dyskinesia is amantadine [86]. However, it should be closely monitored due to the significant number of side effects caused by the higher doses of this noncompetitive NMDA antagonist. Patients with advanced Parkinson’s disease should be referred for the evaluation of possible device-assisted treatment.

## 9. Movement Disorders Associated with Antiseizure Medications

The effects of a single agent on motor symptoms can be therapeutic or toxic, depending on factors such as the dosage, individual differences, concurrent medications, and other unknown conditions. The relationship between antiseizure medications and movement disorders involves various mechanisms, such as the interaction between neurotransmitter systems and the basal ganglia (Table 8). Side effects caused by antiseizure medications depend on various factors, including the type of epilepsy, structural brain changes, and other predisposing factors. However, some patients may experience movement disorders without any preexisting brain diseases. It is common for movement disorders not to receive sufficient attention as potential side effects of antiseizure medications, although reports of such cases have been present since the introduction of anticonvulsants. While it is possible for newer antiseizure medications to also lead to movement disorders, it is likely that these cases are not being fully recognized. Moreover, the factors that may increase an individual’s risk of developing or exacerbating movement disorders due to antiseizure medications are poorly comprehended.

## 10. Movement Disorders Associated with Illicit Drugs

Various abused substances may result in different abnormal movements through their interactions with neurotransmitter systems, such as the dopaminergic, noradrenergic, serotonergic, and GABAergic systems (Table 9) [108]. These aberrant motions may be temporary or persistent, depending on the particular substance, and they may result directly from their sporadic use, abuse, and/or withdrawal. Individual case reports and brief observational case series are the primary sources of toxicity data. Furthermore, adulterants in drugs of abuse, added to increase weight, enhance or imitate a pharmacological action, or improve drug transport, may induce movement disorders. For example, heroin is combined with the synthetic opioid fentanyl hydrochloride, cocaine with diltiazem, methylephedrine with ecstasy and pseudoephedrine, and dextromethorphan with caffeine [109].

Caffeine and pseudoephedrine can cause postural tremors that closely mimic essential tremors [118]. Cocaine may inhibit dopamine reuptake, enhancing dopaminergic drive [119]. Amphetamines cause more widespread catecholaminergic stimulation, but long-term use depletes dopamine and may damage the nigro-striatal neurons [120]. It is well known that the drug 3,4-methylenedioxymethamphetamine (MDMA) causes parkinsonism and serotoninergic syndrome alike [121]. While the movement issue commonly occurs following drug administration, it can also occur during withdrawal. Usually, it improves with the discontinuation of the medication, but the motor symptoms might continue for months. Movement problems brought about by illegal drug usage do not have a particular therapy.

The pathways regulated by psychostimulants and those involved in the pathophysiology of various movement disorders overlap significantly. Movement disorders, which present as hypokinetic or hyperkinetic disorders such as parkinsonism, tremor, dyskinesias, and myoclonus, impact the control of voluntary and involuntary movements. Most of these illnesses directly or indirectly affect basal ganglia circuits. There is additional evidence of abnormal cortical function, white matter tract involvement, and extensive neural network failure, in the development of movement disorders secondary to illicit drugs [122].

## 11. Future Studies

There are important areas to study regarding drug-induced movement disorders. The majority of the medication classes have not been systematically reviewed regarding these abnormalities. Authors reporting movement disorders secondary to drugs should provide more specific details of the clinical history, especially regarding long-term follow-up. The assessment of the patient in the follow-up could demonstrate significant risk factors that should be observed in individuals with abnormal involuntary movements induced by medications. Moreover, they could provide clinical characteristics assisting in the prognosis of neurodegenerative diseases, as in the case of drug-induced parkinsonism, where patients have been observed to present years later with Parkinson’s disease. Myoclonic reports should provide electroencephalogram and surface electromyography descriptions to assess the myoclonic neuroanatomical source. Some authors have already studied the importance of having at least one movement disorder specialist among the authors reporting drug-induced movement disorders, to ensure the quality and authenticity of the description of the clinical picture.

An important benefit of studying drug-induced movement disorders is in the design of new drugs and algorithms to improve clinical practice. Some characteristics among different classes of medications or even the drugs within these classes are distinguished predictors of specific movement disorders. Previous knowledge of these characteristics, associated with the epidemiological profiles of the individuals more frequently affected by these movements, can help the prescriber to choose the most effective medication with fewer side effects.

## 12. Limitations

There are some limitations in the studies of drug-induced movement disorders. The majority of the reports regarding movement disorders secondary to drugs come from case reports and case series. Moreover, when large samples are described, the authors do not provide specific information about the individuals, which can lead to misunderstandings of the main movement disorder. The present manuscript did not perform a systematic approach, which could have led to study selection bias.

## 13. Conclusions

Movement disorders are a frequent and occasionally serious side effect of numerous medications, often dopamine receptor blockers. It might be difficult to pinpoint the responsible substance since patients frequently take mixtures of medications that can lead to many movement disorders. Understanding the common movement abnormalities and their usual clinical courses is necessary for the correct diagnosis. This is important since eliminating the offending drug, with or without adding another pharmacological therapy, is an essential therapeutic strategy for most drug-induced movement disorders.

## Figures and Tables

**Table 1:** Classification of drug-induced movement disorders.

Movement Disorder	DSM V TR, 2022 ^a^	ICD-10 Version 2019 ^b^		ICD-11 Version 2023 ^c^	
		Code	Description	Code	Description
Akathisia	Medication-induced acute akathisia	G25.8	Other specified extrapyramidal and movement disorders (drug-induced akathisia)	8A07.1	Akathisia
Ataxia	NA	R27.0	Ataxia, unspecified	8A03.3Y	Other specified acquired ataxia
Dyskinesia	Tardive dyskinesia	NA	NA	8A01.16	Drug-induced chorea
	NA	G25.4	Drug-induced chorea	8A01.16	Drug-induced chorea
	NA	G25.5	Other chorea	8A01.20	Hemichorea
	NA			8A01.21	Ballism
	NA			8A01.22	Hemiballism
Dystonia	Medication-induced acute dystonia	G24.0	Drug-induced dystonia	8A02.10	Drug-induced dystonia
	Tardive dystonia				
Myoclonus	NA	G25.3	Myoclonus (drug-induced myoclonus)	8A06.Y	Other specified myoclonic disorders
Neuroleptic malignant syndrome	Neuroleptic malignant syndrome	G21.0	Malignant neuroleptic syndrome	8A0Y	Other specified movement disorders
Parkinsonism	Medication-induced parkinsonism	G21.1	Other drug-induced secondary parkinsonism	8A00.24	Drug-induced parkinsonism
	NA	G21.2	Secondary parkinsonism due to other external agents	8A00.2Y	Other specified secondary parkinsonism
Tics	NA	G25.6	Drug-induced tics and other tics of organic origin	8A05.1Y	Other specified secondary tics
Tremor	NA	G25.1	Drug-induced tremor	8A04.31	Tremor due to chronic or acute substance use
	Medication-induced postural tremor	NA	NA	8A04.32	Tremor due to drug withdrawal
	NA	NA	NA	8A04.3Y	Other specified secondary tremor
Other	Other medication-induced movement disorder	NA	NA	NA	NA

Abbreviations: DSM V TR, The Diagnostic and Statistical Manual of Mental Disorders, Fifth Edition, Text Revision; ICD, International Statistical Classification of Diseases and Related Health Problems; NA, not available/not applicable. ^a^ https://www.appi.org/products/dsm (accessed on 16 July 2023). ^b^ https://icd.who.int/browse10/2019/en (accessed on 16 July 2023). ^c^ https://icd.who.int/browse11/l-m/en (accessed on 16 July 2023).

**Table 2:** FreeText and MeSH search terms in the US National Library of Medicine.

Query	Search Terms	Results
drug-induced (AND) movement disorder	“drug-induced” [All Fields] AND (“movement disorders” [MeSH Terms] OR (“movement” [All Fields] AND “disorders” [All Fields]) OR “movement disorders” [All Fields] OR (“movement” [All Fields] AND “disorder” [All Fields]) OR “movement disorder” [All Fields])	9756

**Table 3:** Drug-induced dystonia incidence and levels of evidence [45].

Class of Medication	Incidence	Pathophysiological Mechanism	Level of Evidence
Antipsychotics	1.4–16.5%	Dopamine receptor blocker	A/B
Antiemetics (metoclopramide)	0.2%	Dopamine receptor blocker	A
Selective serotonin reuptake inhibitors	NA	5-HT2 receptor hyperstimulation, inhibition of dopaminergic activity	C
Tricyclic antidepressants	NA	Serotoninergic pathway in the striatum	C
Selective serotonin–norepinephrine reuptake inhibitors	NA	Serotoninergic pathway in the striatum	C
Antiseizure medications	NA	Dopaminergic and GABAergic hypothesis	C
Cholinesterase inhibitors	NA	Cholinergic hypothesis	C
Others: lamivudine, metformin, and albendazole	NA	Variable	C

Abbreviations: NA, not available/not applicable.

**Table 4:** Clinical features of cortical and subcortical myoclonus.

Characteristic	Cortical	Subcortical
Timing	Rhythmic, can be irregular	Periodic
Distribution	Focal, multifocal, generalized	Axial, segmental
Location	Distal	Proximal and distal
Muscle group	One synergistic group	Agonist–antagonist co-contraction
Response to stimulus	Highly sensitive	Not sensitive
Electroencephalogram	Variable, may show epileptiform discharges and slow waves	No consistent abnormalities
Electromyography	Bursts typically less than 75 ms	Variable burst properties; burst duration varies among subtypes
Back-averaged electroencephalogram transients	Variable, but focal sharp wave; time-locked	No correlate; not time-locked
Somatosensory evoked potential ^a^	Giant; enlarged cortical component common	Normal amplitude
Long latency electromyography reflex response	Variable; enhanced long latency reflex (C reflex) typical with cortical reflex myoclonus	Some cases have reflex response to sound
Management	Benzodiazepine-responsive	Benzodiazepine-responsive

^a^ Somatosensory evoked potential, or SEEPs.

**Table 5:** Clinical features of idiopathic Parkinson’s disease and drug-induced parkinsonism.

Feature	Idiopathic Parkinson’s Disease	Drug-Induced Parkinsonism
Onset	Chronic	Acute–subacute
Age at onset	Mean age at sixth decade	More common in elderly
Sex	More common in males	More common in females
Symmetry of clinical manifestations	Unilateral or asymmetric	Often bilateral or symmetric
Lower/upper body involvement	Both	More severe involvement of the upper extremities; also, more hypomimia than gait disorder
Freezing	Common	Uncommon
Glabellar tap	Frequently positive	Infrequently positive
Tremor	Variable, commonly resting	Variable, commonly postural
Rigidity, bradykinesia, and resting tremor	Bradykinesia is a cardinal finding	The most common finding is rigidity
Depression	Common	Common
Dementia	Rare at onset, frequent with neurodegenerative progression	May be present before onset of parkinsonism; rarely, it is caused by the offending drug
Clinical response to dopaminergic drugs	Marked	Poor
Clinical response to anticholinergic drugs	Evident	Mild to moderate
Clinical response to offending drug discontinuation	Poor	Variable
Cerebrospinal fluid levels of homovanillic acid	Low, normal	High
Dopamine transporter scan (DaTscan)	Normal	Abnormal
Positron emission tomography (PET) and single photon emission computed tomography (SPECT) neuroimaging	Reduced uptake of presynaptic markers, normal uptake of dopamine receptor ligands	Normal uptake of presynaptic markers, reduced uptake of dopamine receptor ligands

**Table 6:** Drug-induced parkinsonism-like emergencies.

Feature	Parkinsonism-Hyperpyrexia Disorder	Serotonin Syndrome	Neuroleptic Malignant Syndrome
Incidence	0.3–3.6% of patients with Parkinson’s disease	0.07–0.09% among the patients receiving serotoninergic drugs	0.2% with first-generation antipsychotics; 0.006% with secondary-generation antipsychotics
Mortality	15%	5%	10–20% with first-generation antipsychotics; 5.5% with second-generation antipsychotics
Offending drugs	Dopamine deficit, discontinuation of dopaminergic drugs	Serotoninergic agents and drugs of abuse	Dopamine antagonists or discontinuation of dopamine agonists
Abnormal movement onset after drug initiation	Hours to days	Hours	Days to weeks
Mental status	Catatonia, mutism	Confused, agitated	Catatonia, mutism
Clinical manifestations	Lead-pipe rigidity, hyporreflexia	Myoclonus, tremor, hyperreflexia, muscle rigidity	Lead-pipe rigidity, hyporreflexia
Clinical vital signs	Mild hyperthermia	Autonomic instability	Mild hyperthermia
Management	Reintroduction of dopaminergic treatment or re-implantation of deep brain stimulation	Cessation of triggering agent; serotoninergic antagonists	Cessation of triggering agent; dantrolene

**Table 7:** Pathophysiology of drug-induced tremors.

Medication Class	Tremor			Mechanism	Reference
	Action/Postural	Intention	Resting		
Antiarrhythmics	Amiodarone	NA	Amiodarone	Enhanced physiological tremor, hyperthyroidism	[65]
Antidepressants	Amitriptyline, SSRIs, SNRIs	NA	SSRIs, SNRIs	Enhanced physiological tremor, mainly central component	[66]
Antipsychotics	Haloperidol	NA	Haloperidol	Striatal dopamine receptor blocking	[67]
Antiseizure medication	Valproate		Valproate	Enhanced physiological tremor, dopaminergic dysfunction	[68]
Beta-adrenergic agonists	Albuterol, salmeterol	NA	Albuterol, salmeterol	Enhanced physiological tremor, mechanical reflex component	[69]
Chemotherapy	Cytarabine	Cytarabine	NA	Cerebellar toxicity to Purkinje cells	[70]
Drugs of abuse	Cocaine, ethanol	Ethanol	Cocaine, ethanol	Enhanced physiological tremor, cerebellar toxicity	[71]
Gastrointestinal drugs	Metoclopramide	NA	Metoclopramide, promethazine	Striatal dopamine receptor blocking	[72]
Hormones	Thyroxine, epinephrine	Thyroxine, epinephrine	NA	Enhanced physiological tremor	[73]
Immunosuppressive drugs	Tacrolimus, cyclosporine	Tacrolimus, cyclosporine	NA	Enhanced physiological tremor, specially peripheral component	[74]
Methylxantines	Theophylline	NA	NA	Enhanced physiological tremor	[75]
Mood stabilizers	Lithium	Lithium	Lithium	Enhanced physiological tremor	[76]

Abbreviations: NA, not available/not applicable; SNRI, serotonin–norepinephrine reuptake inhibitor; SSRI, selective serotonin reuptake inhibitor.

**Table 8:** Antiseizure medications associated with movement disorders.

Drug	Akathisia	Ataxia	Dyskinesia	Dystonia	Myoclonus	Parkinsonism	Restless Leg Symptoms	Tics	Tremor	Reference
Brivaracetam		+								[87]
Carbamazepine	+	+	+	+	+	+		+	+	[88]
Cenobamate		+							+	[89]
Clonazepam		+						+	+	[87]
Eslicarbazepine		+							+	[88]
Ethosuximide	+	+	+							[90]
Felbamate	+	+	+	+						[91]
Gabapentin	+	+	+	+	+	+			+	[92]
Lacosamide		+								[93]
Lamotrigine	+	+	+	+	+	+		+	+	[94]
Levetiracetam		+				+				[95]
Oxcarbazepine		+		+	+	+			+	[88]
Perampanel		+								[96]
Phenobarbital		+								[97]
Phenytoin		+	+	+	+	+	+	+	+	[98]
Pregabalin	+	+	+	+	+	+	+		+	[99]
Primidone		+								[100]
Rufinamide		+								[101]
Stiripentol		+								[102]
Tiagabine		+		+						[103]
Topiramate		+	+	+	+		+		+	[104]
Valproate	+	+	+	+	+	+		+	+	[105]
Vigabatrin		+			+	+				[106]
Zonisamide		+					+		+	[107]

Abbreviations: +, at least one report of a movement disorder associated with antiseizure medication.

**Table 9:** Movement disorders associated with psychostimulant drugs.

Psychostimulant Drug	Pathophysyological Mechanism	Movement Disorder	Reference
Amphetamine	Dopamine and serotonin	Ataxia, dyskinesia, dystonia, tremor	[110]
Cathione	Dopamine	Tremor	[111]
Cocaine	Dopamine	Akathisia, dyskinesia, dystonia, myoclonus, parkinsonism, tics, tremor	[112]
Ecstasy (3,4-methylenedioxymethamphetamine)	Dopamine and serotonin	Dystonia, parkinsonism, tremor, restless leg symptoms	[113]
Mephedrone	Dopamine and serotonin	Dyskinesia, myoclonus, tremor	[114]
Methamphetamine	Dopamine and serotonin	Ataxia, dyskinesia, dystonia, tremor	[115]
Methcathinone (ephedrone)	Dopamine and norepinephrine	Dystonia, parkinsonism	[116]
Methylphenidate	Dopamine and norepinephrine	Stereotypical movements	[117]

## Data Availability

Not applicable.
